# RCSB Protein Data Bank: Efficient Searching and Simultaneous Access to One Million Computed Structure Models Alongside the PDB Structures Enabled by Architectural Advances

**DOI:** 10.1016/j.jmb.2023.167994

**Published:** 2023-02-02

**Authors:** Sebastian Bittrich, Charmi Bhikadiya, Chunxiao Bi, Henry Chao, Jose M. Duarte, Shuchismita Dutta, Maryam Fayazi, Jeremy Henry, Igor Khokhriakov, Robert Lowe, Dennis W. Piehl, Joan Segura, Brinda Vallat, Maria Voigt, John D. Westbrook, Stephen K. Burley, Yana Rose

**Affiliations:** 1 - Research Collaboratory for Structural Bioinformatics Protein Data Bank, San Diego Supercomputer Center, University of California, La Jolla, CA 92093, USA; 2 - Research Collaboratory for Structural Bioinformatics Protein Data Bank, Rutgers, The State University of New Jersey, Piscataway, NJ 08854, USA; 3 - Institute for Quantitative Biomedicine, Rutgers, The State University of New Jersey, Piscataway, NJ 08854, USA; 4 - Cancer Institute of New Jersey, Rutgers, The State University of New Jersey, New Brunswick, NJ 08901, USA; 5 - Department of Chemistry and Chemical Biology, Rutgers, The State University of New Jersey, Piscataway, NJ 08854, USA

**Keywords:** FAIR principles, computer architecture, databases, structural biology, protein structure prediction

## Abstract

The Research Collaboratory for Structural Bioinformatics Protein Data Bank (RCSB PDB) provides open access to experimentally-determined three-dimensional (3D) structures of biomolecules. The RCSB PDB RCSB.org research-focused web portal is used annually by many millions of users around the world. They access biostructure information, run complex queries utilizing various search services (*e.g.*, full-text, structural and chemical attribute, chemical, sequence, and structure similarity searches), and visualize macromolecules in 3D, all at no charge and with no limitations on data usage. Notwithstanding more than 24,000-fold growth of the PDB over the past five decades, experimentally-determined structures are only available for a small subset of the millions of proteins of known sequence. Recently developed machine learning software tools can predict 3D structures of proteins at accuracies comparable to lower-resolution experimental methods. The RCSB PDB now provides access to ~1,000,000 Computed Structure Models (CSMs) of proteins coming from AlphaFold DB and the ModelArchive alongside ~200,000 experimentally-determined PDB structures. Both CSMs and PDB structures are available on RCSB.org and *via* well-established RCSB PDB Data, Search, and 1D-Coordinates application programming interfaces (APIs). Simultaneous delivery of PDB data and CSMs provides users with access to complementary structural information across the human proteome and those of model organisms and selected pathogens. API enhancements are backwards-compatible and programmatic users can “opt in” to access CSMs with minimal effort. Herein, we describe modifications to RCSB PDB cyberinfrastructure required to support sixfold scaling of 3D biostructure data delivery and lay the groundwork for scaling to accommodate hundreds of millions of CSMs.

## Introduction

The Research Collaboratory for Structural Bioinformatics (RCSB) Protein Data Bank (PDB) RCSB.org research-focused web portal^1–3^ provides open access to a rigorously validated, expertly biocurated collection of ~200,000 experimentally-determined 3D structures of biological macromolecules stored in the PDB archive.^2,4^ It is used annually by many millions of researchers, educators, and students for a range of general and investigative applications. Users can directly access deposited structures or make use of a modular, yet fully-integrated software suite that supports interrogation, visualization, and analyses of 3D protein structures. The RCSB.org web portal is powered by a recently redesigned, highly-flexible, extensible search and data delivery architecture.^5^ Several application programming interfaces (APIs) support comprehensive search (search.rcsb.org), data delivery (data.rcsb.org), and sequence-focused (1d-coordinates.rcsb.org) functionalities. The cyberinfrastructure architecture was designed to accommodate the ever-growing number of deposited PDB structures, while integrating annotations and other information from ~50 trusted external data resources.^6,7^

The PDB archive stores ~200,000 experimentally-determined structures from more than 50 years of protein structure determination efforts by researchers working on every inhabited continent. Notwithstanding this impressive metric, there are in fact no experimentally-determined 3D structure data for most of the >200 million protein sequences represented in UniProt.^8^ This chasm between known protein sequences and available 3D biostructures continues to widen despite advances in cryo-electron microscopy,^9,10^ *etc*.

For more than 50 years, scientists have tried to elucidate the relationship between protein sequence and structure to predict protein structures directly from their amino acid sequence. Several key discoveries^11–15^ culminated in the development of AlphaFold2^16^ by John Jumper and co-workers, which utilizes sequence and 3D structure information, coevolution data, and machine learning (ML) methods. The success of AlphaFold2 in predicting individual protein structures was announced at CASP14 (Critical Assessment of protein Structure Prediction).^17^ RoseTTAFold,^18^ developed subsequently by David Baker and co-workers, supports prediction of both individual protein structures and those of binary complexes, exemplified with 1,106 heterodimers of *Saccharomyces cerevisiae* proteins.^19^

Recognizing the benefits of whole proteome coverage, RCSB PDB decided to integrate the first three AlphaFold DB (alphafold.ebi.ac.uk)^10^ releases (July 2021, December 2021, and January 2022) and selected RoseTTAFold predictions stored in the ModelArchive (modelarchive.org)^20^ into RCSB.org. Doing so, allowed users to explore and analyze ~1 million Computed Structure Models (CSMs) alongside experimentally-determined structures.^3^ Enhancement of RCSB.org with CSMs is particularly useful for proteins of interest not present in the PDB. Several powerful search services allow users to detect similarities between CSMs and experimentally-determined PDB structures, facilitating transfer of annotations and knowledge available for the set of rigorously-validated and expertly-biocurated PDB structures to (currently) annotation-sparse CSMs. Since the advent of AlphaFold2 we have seen advances in multimeric structure prediction^21^ and those without recourse to co-evolutionary information.^22,23^ For currently available CSMs, confidently predicted polypeptide chain segments are comparable in accuracy to lower-resolution experimental structures (*i.e.*, worse than 3.5 Å resolution).^17,24^

Herein, we present recent upgrades to RCSB.org architecture (compared to our previous report in 5 and demonstrate how APIs can be leveraged to search for and programmatically retrieve data on ~1 million CSMs from AlphaFold DB and ModelArchive. A related publication^3^ describes changes to the RCSB.org user interface (UI) and showcases how users can interrogate CSMs with a plethora of browser-based tools.

The following design objectives were established for integration of CSMs into the RCSB.org web portal:

1. Integrate CSM data as “first class objects” and treat them just like experimentally-determined PDB structures within RCSB PDB APIs. Doing so enables seamless integration of CSMs into existing cyber infrastructure. This requirement also means that CSM data are propagated throughout the entire data architecture and that all RCSB.org tools will work with CSMs.
2. Maintain provenance, allowing clear differentiation between experimentally-determined PDB structures and CSMs. Notwithstanding tight integration (prime objective), it must be clear when atomic coordinates shown within the UI or returned by an API describe experimentally-determined PDB structures or CSMs.
3. Extend RCSB PDB adherence to the FAIR principles (Findability Accessibility Interoperability Reusability) to CSMs, and cross-reference CSM resources in the same way as for PDB data.

## Methods

### Overview of existing architecture

**Figure 1.**
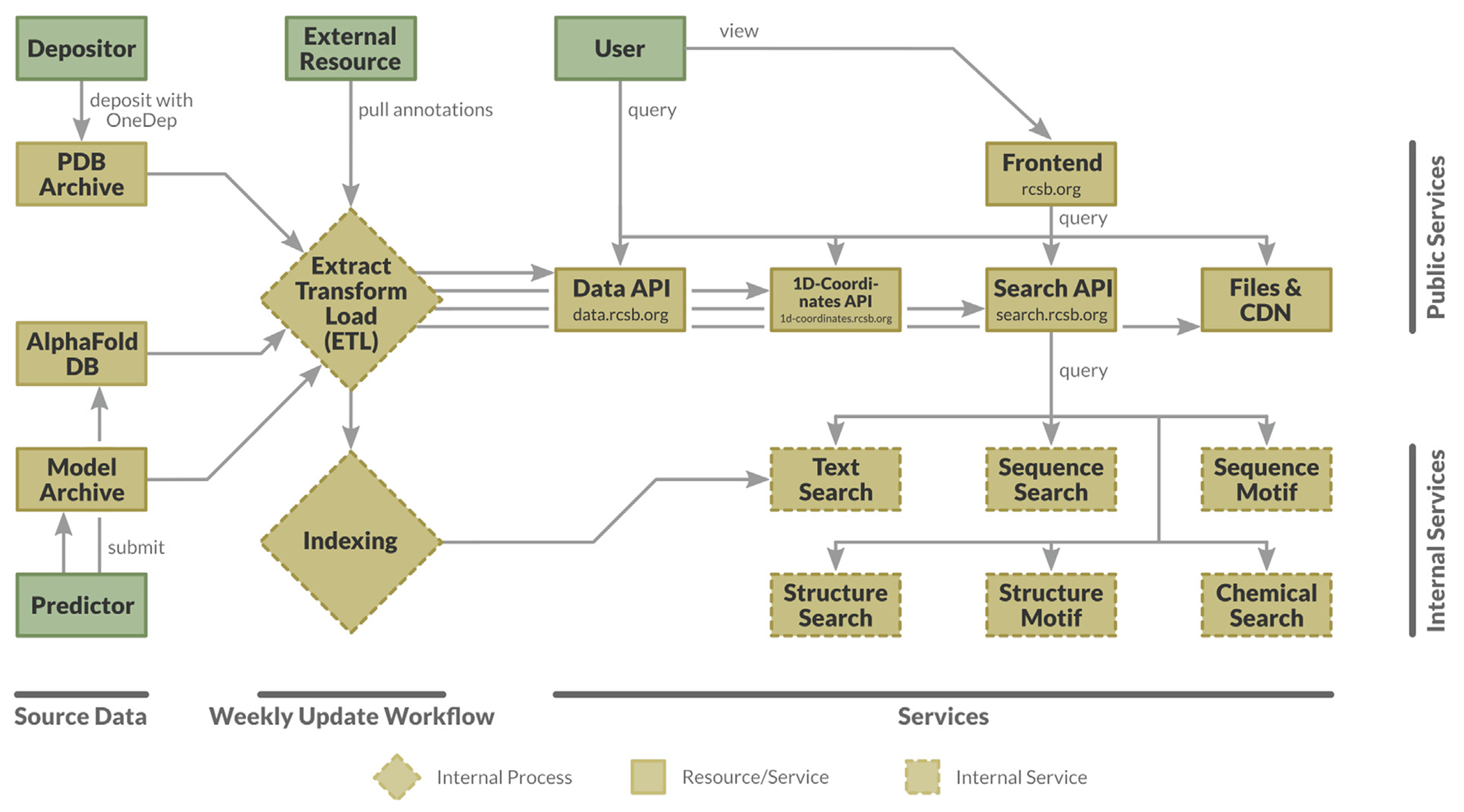
Architectural overview of the RCSB.org web portal. Support for CSMs coming from AlphaFold DB and the ModelArchive was added in September 2022.

The research-focused RCSB.org web portal is built on a modular microservice architecture (depicted on the right-hand side of Figure 1). Most users will only interact with the frontend service, accessible from RCSB.org. The frontend requests static files, such as 3D structure information, from dedicated endpoints or a content delivery network (CDN, cdn.rcsb.org). More complex data (*e.g.*, per-structure meta-information or mappings between different flavors of identifiers) can be requested *via* the Data API (data.rcsb.org). Search requests are processed by the Search API (search.rcsb.org), which delegates subqueries to dedicated internal search services and aggregates individual query responses into a single query response. Doing so effectively separates responsibilities of the RCSB PDB search aggregator and actual search implementations, allowing RCSB.org architecture to follow the single responsibility principle. Expert users can directly interact with Data API, 1D-Coordinates API, Search API, and/or static data. The entire frontend was built upon public RCSB.org services.

Individual services can be accessed using REST (representational state transfer) and/or GraphQL (graphql.org). All services are run on geo-redundant cyber infrastructure located in bicoastal data centers at Rutgers, The State University of New Jersey and the University of California San Diego. A global DNS (domain name system) directs users to the nearest available data center.

Data delivered by RCSB.org are updated weekly. Every Wednesday, at 00:00 Universal Time Coordinated (UTC), several hundred new experimentally-determined PDB structures are publicly released. These new PDB structures originate from depositions made *via* the Worldwide Protein Data Bank (wwPDB)^25^ OneDep software system.^26^ They are rigorously validated and expertly biocurated by one of the wwPDB data centers (depositions coming from the Americas & Oceania → RCSB PDB; Europe and Africa → Protein Data Bank in Europe; Asia and the Middle East → Protein Data Bank Japan). Every Friday, an ETL (extract transform load) pipeline processes all new 3D biostructure data and enriches it with annotations from ~50 external resources.^6,7^ Several search indices are created to facilitate different query styles behind the Search API (*e.g.*, queries by free text, attributes, or identifiers).

### Schema changes to support integration of computed structure models

The RCSB.org web portal follows a schema-first approach. This single source of truth simplifies software development, prevents inconsistencies, and improves code maintainability. Source files in mmCIF format contain a plethora of important annotations and metadata. This information is propagated to each service, leveraging the PDBx/mmCIF (Protein Data Bank Exchange (PDBx) macromolecular Crystallographic Information Framework (mmCIF)) data dictionary^27^ and a compatible, private schema used by all RCSB.org services. A wwPDB PDBx/mmCIF Working Group manages the data dictionary in collaboration with wwPDB members. Their deliberations and data dictionary content are published on GitHub (github.-com/pdbxmmcifwg) and a data portal site (mmcif.wwpdb.org), respectively. ModelCIF is a flexible CSM-focused dictionary extension of the PDBx/mmCIF data standard which was developed jointly for use with CSMs by the wwPDB and stakeholders drawn from the structure prediction community. CSMs from AlphaFold DB and RoseTTAFold-generated CSMs (freely available *via* ModelArchive) conform to the ModelCIF standard,^28^ which both improved data integrity and reduced efforts required to integrate additional properties or attributes (such as CSM-specific quality assessment categories) into the RCSB.org cyberinfrastructure. The Model-CIF data dictionary is available at github.com/ihmwg/ModelCIF. Frameworks describing the PDBx/mmCIF and ModelCIF dictionaries are regulated by Dictionary Definition Language 2 (DDL2), a generic language that supports construction of dictionaries composed of data items grouped into categories.^29^ DDL2 supports primary data types (*e.g.*, integers, real numbers and text), boundary conditions, controlled vocabularies, and linking of data items together to express relationships (*e.g.*, parent–child related data items). DDL2 is described by its own dictionary and is, therefore, self-validating.

Some additions were made to the RCSB.org internal schema (which can be explored using the top-right “Docs” button at data.rcsb.org/graphql/index.html). For example, we introduced the “rcsb_comp_model_provenance” category, which tracks provenance of each CSM, and the “rcsb_ma_qa_metric_global” that captures the overall quality of the CSM prediction. Both AlphaFold2 and RoseTTAFold predictions include pLDDT values (predicted local distance difference test; original lDDT score is defined in 30). In addition, changes were made to labels of certain data properties appearing in APIs and the UI, in order to avoid property names that would be misleading in the context of CSMs. For example, “PDB ID” was rebranded as “Entry ID”, “Deposition” changed to “Structure Details”, and “Deposition Author” was renamed to “Structure Author”.

### Integrating computed structure models from repositories

The source of AlphaFold2 predictions is AlphaFold DB (alphafold.ebi.ac.uk).^10^ Its first release in July 2021 encompassed ~360,000 CSMs covering proteomes of 21 model organisms. In December 2021, a second set of predictions for SwissProt database sequences^31^ was released, and predictions covering 32 additional proteomes relevant to global health were released in January 2022. MANE predictions (Matched Annotation from NCBI and EMBL-EBI) were also integrated. These AlphaFold2 predictions number 999,255 (excluding redundancies), all of which are now integrated into RCSB.org. Additionally, we included 1,106 RoseTTAFold predictions,^19^ stored in the ModelArchive (modelarchive.org). More than 200 million AlphaFold2 CSMs were released in July 2022. Their integration into RCSB.org is underway.

Local copies of the ModelCIF files were created for both AlphaFold DB and ModelArchive CSMs, so that underlying structure files could be normalized as needed to ensure full compatibility with existing cyberinfrastructure. Local storage reduces strain on upstream repositories, circumvents possible connectivity issues when pulling data from external resources, and enables better overall performance. Newer versions of existing files and/or additional CSMs can be pulled from upstream repositories whenever it is warranted to do so.

### Establishing unique identifiers

AlphaFold DB and the ModelArchive provide FAIR-compatible identifiers, which are namespaced and indicate source repository (*e.g*., “AFA0A452S449-F1” from AlphaFold DB and “ma-bak-cepc-0001” from ModelArchive). There is, however, no community-wide standard to enforce this convention of name-spaced identifiers, composed of alphanumeric characters and dashes (“–”). And there is no guarantee that identifiers introduced by other CSM repositories would be unique and/or only composed of alphanumeric characters and dashes.

Custom identifiers were introduced to normalize entry identifiers during loading into the RCSB PDB Data Warehouse. During the loading process, external identifiers are converted into RCSB.org-specific identifiers, which follow the format “${database}_${identifier}” (*e.g.*, identifier “AFA0A452S449-F1” maps to “AF_AFA0A452S449F1”). This practice aligns with extended PDB ID codes (PDB_00001ABC, rcsb.org/news/607760112786e73a79c76f9d), which will be introduced when all 4-character PDB entry identifiers have been exhausted. In addition, this approach ensures uniqueness, robustness (*i.e.*, protection from non-standard characters), and functionality (*i.e.*, regular expressions can distinguish between experimentally-determined PDB structures and CSMs). We also retain original identifiers to maintain provenance, ensure interoperability with external resources, and support user searches with original identifiers.

### Performance improvements

Weekly updates of the PDB archive typically involve release of information for ~300 new experimentally-determined structures. These data are loaded *via* the Weekly Update Workflow, a process that starts on Fridays and is scheduled to finish no later than 00:00 UTC on Wednesdays, when new PDB data are made public. Sequence clusters are re-computed weekly, including CSM sequences to generate consistent cluster solutions. Every week, indices are recreated for critical information (*e.g.*, entry identifiers, select annotations). Even if source data remained unchanged, external annotations could change from week to week, requiring reindexing. The Weekly Update Workflow runs independently in each of the RCSB PDB bicoastal data centers. More than 100 individual data management tasks are performed within the Workflow, orchestrated by the Luigi framework (github.com/spotify/luigi). Using Luigi worked well for our transition from ~200,000 to >1.2 million structures. Because of the distributed design of Luigi, minimal changes in the RCSB PDB workflow were required to scale it horizontally utilizing additional OpenStack instances (*i.e.*, more CPUs and memory). These process modifications enabled 6-fold speedup of the usual weekly loading process.

Across the entire RCSB PDB Data Warehouse, Elasticsearch (elastic.co) is used for text indexing, while MongoDB (mongodb.com) serves as the database backend of individual services. These enterprise-grade software solutions are readily configurable as distributed systems, facilitating scale up of RCSB PDB operations. A similar strategy can be applied to our scientific search services. For further scaling, data can be divided into shards and distinct instances could process smaller, more manageable data batches. The RCSB PDB microservice-based architecture facilitates scaling of individual services, without affecting others, and allows dynamic deployment of additional instances of resource-strapped services. Scaling would not have been possible with the legacy monolithic RCSB PDB data and software architecture.

## Results

Information for >1 million CSMs is now available *via* the RCSB PDB Data API. Additionally, they can be searched *via* Search API. No “breaking changes” were made and programmatic users can tap into the potential of CSMs with minimal code changes.

### Data API: Access metadata of computed structure models

**Figure 2.**
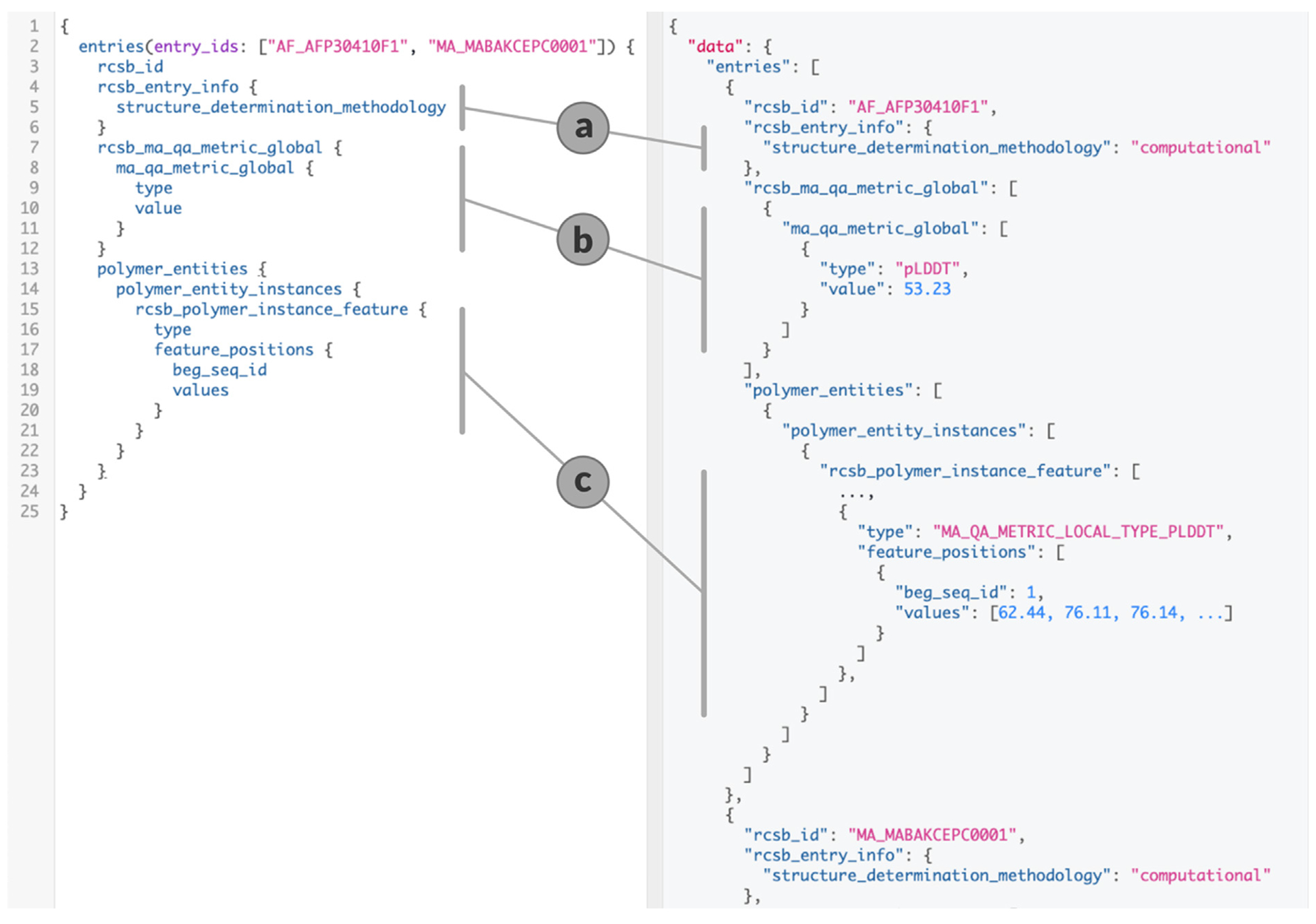
Data API query for CSM metadata on the left, response on the right. (a) Indicates whether this entry is a CSM or a PDB structure. (b) Provides the global pLDDT value. (c) Contains per-residue confidence values.

CSMs share endpoints and query definitions with experimentally-determined PDB structures. Information relating to CSMs can be retrieved by providing the corresponding entry, assembly, entity, or instance identifier. Some properties are not relevant for CSMs (*e.g.*, experimental details, PDB archive deposition details, and listings of wwPDB validation reports). Analogously, AlphaFold2 and RoseTTAFold CSMs possess properties distinct from those of PDB structures (*e.g.*, attributes to describe provenance and pLDDT scores). The GraphQL snippet illustrated in Figure 2 summarizes prominent new CSM properties: “rcsb_entry_info.structure_determination_methodology” delineates experimentally-determined PDB structures and CSMs (Figure 2(a)), “rcsb_ma_qa_metric_global” provides per-structure quality scores (Figure 2(b)). Per-residue prediction confidence scores are available as polymer instance features (Figure 2(c)).

### Search API: Search by properties of computed structure models

**Figure 3.**
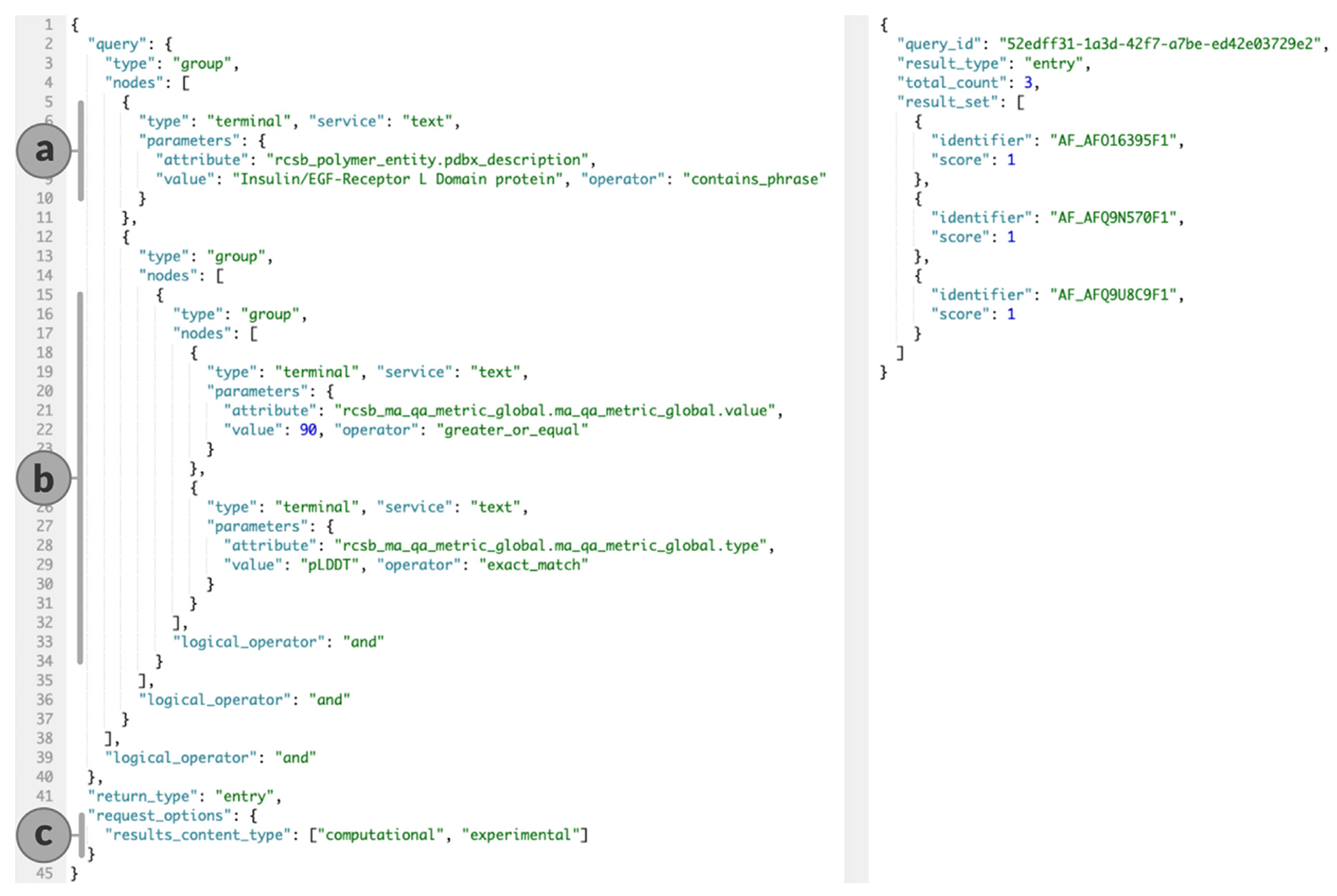
Search API query with predicate based on CSM metadata on the left, response on the right. (a) Text query. (b) Filters for very high pLDDT confidence values >90. (c) Requests CSMs.

The RCSB PDB Search API acts as an aggregator that bundles results from different search services. For example, it is possible to combine a full-text search (Figure 3(a)) with an attribute-based predicate (Figure 3(b)), using the full complement of Boolean operators. Queries do not include CSMs by default (which helps to ensure that programmatic users of RCSB PDB APIs do not face any “breaking changes”). CSMs can be included by specifying “experimental” and “computational” as “results_content_type” property (Figure 3(c)). This property is optional and defaults to “experimental”, excluding CSMs unless explicitly requested. The “rcsb_entry_info.structure_determination_methodology” attribute can be used to build complex queries encompassing different criteria for experimentally-determined PDB structures and CSMs. Range operators (Figure 3(b)) enable filtering for CSMs with very high confidence scores, as indicated by average pLDDT scores >90. The “rcsb_comp_model_provenance.source_db” attribute captures the origin of a CSM and allows filtering for “AlphaFoldDB” or “ModelArchive” entries. By default, experimentally-determined structures are prioritized over CSMs and fine-grained sorting options are available to adjust this behavior.

The Search API depends on several internal scientific search services. An Elasticsearch cluster implements text- and attribute-based searches, allowing users to query by full text (comparable to a Google search) and by predicates on single attributes (*e.g.*, length of a polymer chain between 100 and 120 residues). “Off-the-shelf” implementations are used for chemical similarity (OpenEye Chemical Toolkit, eyesopen.com/oechem-tk) and sequence searching (MMseqs2).^32^ Structure similarity searching is implemented using BioZernike descriptors,^33^ which capture the 3D shape of proteins using 3D Zernike moments and enable efficient comparisons. Specific 3D arrangements of small groups of residues (such as the Serine-Histidine-Aspartate catalytic triad) can be detected using the structure motif search service.^34^ An in-house library based on regular expressions allows searching for sequence motifs. These internal services were adapted to honor the “results_content_types” property, which reduces computational load on internal services if a query is restricted to PDB structures.

### Assessment for computed structure models

Easy access to 3D biostructure quality assessment measures is a critical feature of the RCSB.org web portal.^24,25^ Structure validation is crucial when gauging the potential utility of PDB structures. Experimentally-determined structures are rigorously validated and expertly biocurated by the global wwPDB Biocuration Team at the time of deposition. CSMs lack this “gatekeeping” step and low confidence predictions are inevitable. AlphaFold2 and RosettaFold generate per-residue pLDDT values (see above), which serve as prediction confidence measures.

The Data API provides access to global and per-residue pLDDT values for each integrated CSM and each of its polymer instances, respectively (the first in the dedicated “rcsb_ma_qa_metric_global” category, the second as a new instance feature of type “MA_QA_METRIC_LOCAL_TYPE_PLDDT”). Low confidence regions of CSMs with pLDDT values <70 are ignored by the structure similarity and structure motif search services, which reduces false-positives in the search result set. Users can also sort and filter by average (“global”) pLDDT values of CSMs and further narrow their result set to high confidence predictions, as desired.

### Documentation and help pages

A comprehensive collection of help topics (rcsb.org/docs) describes services and views available on the RCSB.org web portal. Extant documentation was updated to include information for CSMs. In parallel, dedicated help pages pertaining to CSMs were added. Each of the RCSB PDB public services and APIs offers documentation for programmatic users (*e.g.*, search.rcsb.org for Search API and data.rcsb.org for Data API). These resources are regularly updated based on user feedback and questions coming into the RCSB.org Customer Service Help Desk.

### Availability

Referenced backend services are available at search.rcsb.org, data.rcsb.org, and 1d-coordinates.rcsb.org.

Many underlying projects are open-source GitHub projects and can be used as software dependencies. These projects may be of assistance to users adapting codebases for use with CSMs. Public software projects include the Mol* 3D viewer (github.com/molstar/molstar),^35^ its RCSB.org-specific flavor (github.com/molstar/rcsb-molstar), a 2D macromolecular image renderer (github.com/molstar/molrender), the Protein Feature Viewer stack (github.com/rcsb/rcsb-saguaro, github.com/rcsb/rcsb-saguaro-app, github.com/rcsb/rcsb-saguaro-3d),^36,37^ the UI of the Alignment application (github.com/rcsb/rcsb-pecos-app), CIF parsers (github.com/rcsb/ciftools-java, github.com/rcsb/py-mmcif) supporting BinaryCIF^38^ and ModelCIF,^28^ BioJava (github.com/biojava/biojava),^39^ for 3D structure management and analysis, volume/shape-based structure similarity search (github.com/biocryst/biozernike),^33^ and structure motif search (github.com/rcsb/strucmotif-search).^34^

## Discussion

Integration of CSMs within the RCSB.org web portal allows users for the first time to navigate >1 million high-value CSMs coming from AlphaFold DB and ModelArchive. RCSB.org offers unique tools to search by sequence similarity, sequence motif, structure similarity, or structure motif, and detect resemblances between and among CSMs and/or experimentally-determined PDB structures. Several other groups have also made substantial progress when it comes to providing some of these search services.^40–42^ The unique feature of the newly-released version of RCSB.org, however, lies in the fact that every-one of these scientific search services can be accessed in a one-stop-shop. This unified functionality allows users to define complex queries by combining multiple search services (*e.g.*, combining sequence similarity and structure comparison). Updated RCSB PDB APIs provide direct access to the underlying data and require minimal adjustment by programmatic users who want to tap into the enormous potential of >1 million CSMs.

Going forward, our highest priority is to further scale RCSB PDB cyberinfrastructure to integrate the current AlphaFold DB release of ~214 million predictions. In addition, we plan to integrate external annotations specific to CSMs^43,44^ and improve RCSB.org tools available for synchronizing annotations between experimentally-determined PDB structures with CSMs (*e.g.*, by implementing such features in our structure alignment tools). In the longer term, we will solicit input from RCSB PDB data consumers to determine how best to continue making PDB structures available alongside the rapidly-growing corpus of CSMs being made available worldwide.

## Abbreviations:

RCSB PDB: Research Collaboratory for Structural Bioinformatics Protein Data Bank
3D: three-dimensional
CSM: Computed Structure Model
API: Application Programming Interface
ML: Machine Learning
CASP: Critical Assessment of protein Structure Prediction
UI: User Interface
FAIR principles: Findability Accessibility Interoperability Reusability
CDN: Content Delivery Network
REST: REpresentational State Transfer
DNS: Domain Name System
UTC: Universal Time Coordinated
wwPDB: worldwide Protein Data Bank
ETL: Extract Transform Load
DDL2: Dictionary Definition Language 2
pLDDT: predicted Local Distance Difference Test
PDBx/mmCIF: Protein Data Bank Exchange (PDBx) macromolecular Crystallographic Information Framework (mmCIF)

## References

[1] Burley SK, Bhikadiya C, Bi C, Bittrich S, Chen L, Crichlow GV, , (2022). RCSB Protein Data Bank: Celebrating 50 years of the PDB with new tools for understanding and visualizing biological macromolecules in 3D. Protein Sci. 31, 187–208. 34676613 10.1002/pro.4213 PMC8740825

[2] Burley SK, Bhikadiya C, Bi C, Bittrich S, Chen L, Crichlow G, , (2021). RCSB Protein Data Bank: Powerful new tools for exploring 3D structures of biological macromolecules for basic and applied research and education in fundamental biology, biomedicine, biotechnology, bioengineering, and energy sciences. Nucleic Acids Res. 49, D437–D451. 33211854 10.1093/nar/gkaa1038 PMC7779003

[3] Burley SK, Bhikadiya C, Bi C, Bittrich S, Chao H, Chen L, , (2023). RCSB Protein Data Bank (RCSB.org): Delivery of Experimentally-Determined PDB Structures Alongside One Million Computed Structure Models of Proteins from Artificial Intelligence/Machine Learning. Nucleic Acids Res. 51, D488–D508. 36420884 10.1093/nar/gkac1077 PMC9825554

[4] Protein Data Bank, (1971). Crystallography: Protein Data Bank. Nature (London), New Biol. 233, 223.16063295

[5] Rose Y, Duarte JM, Lowe R, Segura J, Bi C, Bhikadiya C, , (2021). RCSB Protein Data Bank: Architectural Advances Towards Integrated Searching and Efficient Access to Macromolecular Structure Data from the PDB Archive. J. Mol. Biol 433, 166704 33186584 10.1016/j.jmb.2020.11.003 PMC9093041

[6] Burley SK, Berman HM, Duarte JM, Feng Z, Flatt JW, Hudson BP, , (2022). Protein Data Bank: A Comprehensive Review of 3D Structure Holdings and Worldwide Utilization by Researchers, Educators, and Students. Biomolecules 12, 1425. 36291635 10.3390/biom12101425 PMC9599165

[7] Bittrich S, Rose Y, Segura J, Lowe R, Westbrook J, Duarte JM, , (2022). RCSB Protein Data Bank: Improved Annotation, Search, and Visualization of Membrane Protein Structures Archived in the PDB. Bioinformatics 38, 1452–1454. 34864908 10.1093/bioinformatics/btab813 PMC8826025

[8] UniProt Consortium, (2021). UniProt: the universal protein knowledgebase in 2021. Nucleic Acids Res. 49, D480–D489. 33237286 10.1093/nar/gkaa1100 PMC7778908

[9] de Oliveira TM, van Beek L, Shilliday F, Debreczeni JÉ, Phillips C, (2021). Cryo-EM: the resolution revolution and drug discovery. SLAS Discovery 26, 17–31. 33016175 10.1177/2472555220960401

[10] Varadi M, Anyango S, Deshpande M, Nair S, Natassia C, Yordanova G, , (2022). AlphaFold Protein Structure Database: massively expanding the structural coverage of protein-sequence space with high-accuracy models. Nucleic Acids Res. 50, D439–D444. 34791371 10.1093/nar/gkab1061 PMC8728224

[11] Göbel U, Sander C, Schneider R, Valencia A, (1994). Correlated mutations and residue contacts in proteins. Proteins: Struct. Funct. Bioinf 18, 309–317. 10.1002/prot.340180402 8208723

[12] Rost B, Sander C, (1994). Combining evolutionary information and neural networks to predict protein secondary structure. Proteins: Struct. Funct. Bioinf 19, 55–72. 10.1002/prot.340190108 8066087

[13] Marks DS, Colwell LJ, Sheridan R, Hopf TA, Pagnani A, Zecchina R, , (2011). Protein 3D structure computed from evolutionary sequence variation. PLoS One 6, e28766. 22163331 10.1371/journal.pone.0028766 PMC3233603

[14] Garnier J, Osguthorpe DJ, Robson B, (1978). Analysis of the accuracy and implications of simple methods for predicting the secondary structure of globular proteins. J. Mol. Biol 120, 97–120. 642007 10.1016/0022-2836(78)90297-8

[15] Chothia C, Lesk AM, (1986). The relation between the divergence of sequence and structure in proteins. EMBO J. 5, 823–826. 3709526 10.1002/j.1460-2075.1986.tb04288.x PMC1166865

[16] Jumper J, Evans R, Pritzel A, Green T, Figurnov M, Ronneberger O, , (2021). Highly accurate protein structure prediction with AlphaFold. Nature 596, 583–589. 34265844 10.1038/s41586-021-03819-2 PMC8371605

[17] Kryshtafovych A, Schwede T, Topf M, Fidelis K, Moult J, (2021). Critical assessment of methods of protein structure prediction (CASP)-Round XIV. Proteins: Struct. Funct. Bioinf 89, 1607–1617. 10.1002/prot.26237 PMC8726744 34533838

[18] Baek M, DiMaio F, Anishchenko I, Dauparas J, Ovchinnikov S, Lee GR, , (2021). Accurate prediction of protein structures and interactions using a three-track neural network. Science 373, 871–876. 34282049 10.1126/science.abj8754 PMC7612213

[19] Humphreys IR, Pei J, Baek M, Krishnakumar A, Anishchenko I, Ovchinnikov S, , (2021). Computed structures of core eukaryotic protein complexes. Science 374, eabm4805. 34762488 10.1126/science.abm4805 PMC7612107

[20] Schwede T, Sali A, Honig B, Levitt M, Berman HM, Jones D, , (2009). Outcome of a workshop on applications of protein models in biomedical research. Structure 17, 151–159. 19217386 10.1016/j.str.2008.12.014 PMC2739730

[21] Evans R, O’Neill M, Pritzel A, Antropova N, Senior A, Green T, , (2022). Protein complex prediction with AlphaFold-Multimer. bioRxiv. 10.1101/2021.10.04.463034.

[22] Wu R, Ding F, Wang R, Shen R, Zhang X, Luo S, , (2022). High-resolution de novo structure prediction from primary sequence. bioRxiv. 10.1101/2022.07.21.500999.

[23] Lin Z, Akin H, Rao R, Hie B, Zhu Z, Lu W, , (2022). Evolutionary-scale prediction of atomic level protein structure with a language model. bioRxiv. 10.1101/2022.07.20.500902. 36927031

[24] Shao C, Bittrich S, Wang S, Burley SK, (2022). Assessing PDB Macromolecular Crystal Structure Confidence at the Individual Amino Acid Residue Level. Structure 30, 1385–1394. 36049478 10.1016/j.str.2022.08.004 PMC9547844

[25] wwPDB consortium, (2019). Protein Data Bank: the single global archive for 3D macromolecular structure data. Nucleic Acids Res. 47, D520–D528. 30357364 10.1093/nar/gky949 PMC6324056

[26] Young JY, Westbrook JD, Feng Z, Sala R, Peisach E, Oldfield TJ, , (2017). OneDep: Unified wwPDB System for Deposition, Biocuration, and Validation of Macromolecular Structures in the PDB Archive. Structure 25, 536–545. 28190782 10.1016/j.str.2017.01.004 PMC5360273

[27] Westbrook JD, Young JY, Shao C, Feng Z, Guranovic V, Lawson C, , (2022). PDBx/mmCIF Ecosystem: Foundational semantic tools for structural biology. J. Mol. Biol 434, 167599 35460671 10.1016/j.jmb.2022.167599 PMC10292674

[28] Vallat B, Tauriello G, Bienert S, Haas J, Webb BM, Zidek A, , (2022). ModelCIF: An extension of PDBx/mmCIF data representation for computed structure models. bioRxiv. 10.1101/2022.12.06.51855. PMC10293049 36828268

[29] Westbrook JD, Berman HM, Hall SR, (2005). 2.6 Specification of a relational Dictionary Definition Language (DDL2). In: Hall SR, McMahon B (Eds.), International Tables for Crystallography. Springer, Dordrecht, The Netherlands, pp. 61–72.

[30] Mariani V, Biasini M, Barbato A, Schwede T, (2013). lDDT: a local superposition-free score for comparing protein structures and models using distance difference tests. Bioinformatics 29, 2722–2728. 23986568 10.1093/bioinformatics/btt473 PMC3799472

[31] Boeckmann B, Bairoch A, Apweiler R, Blatter M-C, Estreicher A, Gasteiger E, , (2003). The SWISS-PROT protein knowledgebase and its supplement TrEMBL in 2003. Nucleic Acids Res. 31, 365–370. 12520024 10.1093/nar/gkg095 PMC165542

[32] Steinegger M, Soding J, (2017). MMseqs2 enables sensitive protein sequence searching for the analysis of massive data sets. Nat. Biotechnol 35, 1026–1028. 29035372 10.1038/nbt.3988

[33] Guzenko D, Burley SK, Duarte JM, (2020). Real time structural search of the Protein Data Bank. PLoS Comput. Biol 16, e1007970. 32639954 10.1371/journal.pcbi.1007970 PMC7371193

[34] Bittrich S, Burley SK, Rose AS, (2020). Real-time structural motif searching in proteins using an inverted index strategy. PLoS Comput. Biol 16, e1008502. 33284792 10.1371/journal.pcbi.1008502 PMC7746303

[35] Sehnal D, Bittrich S, Deshpande M, Svobodova R, Berka K, Bazgier V, , (2021). Mol* Viewer: modern web app for 3D visualization and analysis of large biomolecular structures. Nucleic Acids Res. 49, W431–W437. 33956157 10.1093/nar/gkab314 PMC8262734

[36] Segura J, Rose Y, Bittrich S, Burley SK, Duarte JM, (2022). RCSB Protein Data Bank 1D3D module: Displaying positional features on macromolecular assemblies. Bioinformatics 38, 3304–3305. 35543462 10.1093/bioinformatics/btac317 PMC9191206

[37] Segura J, Rose Y, Westbrook J, Burley SK, Duarte JM, (2020). RCSB Protein Data Bank 1D tools and services. Bioinformatics 36, 5526–5527. 10.1093/bioinformatics/btaa1012 PMC8016458 33313665

[38] Sehnal D, Bittrich S, Velankar S, Koča J, Svobodová R, Burley SK, , (2020). BinaryCIF and CIFTools—Lightweight, Efficient and Extensible Macromolecular Data Management. PLoS Comput. Biol 16, e1008247. 33075050 10.1371/journal.pcbi.1008247 PMC7595629

[39] Lafita A, Bliven S, Prlic A, Guzenko D, Rose PW, Bradley A, , (2019). BioJava 5: A community driven open-source bioinformatics library. PLoS Comput. Biol 15, e1006791. 30735498 10.1371/journal.pcbi.1006791 PMC6383946

[40] van Kempen M, Kim SS, Tumescheit C, Mirdita M, Söding J, Steinegger M, (2022). Foldseek: fast and accurate protein structure search. bioRxiv. 10.1101/2022.02.07.479398. PMC10869269 37156916

[41] Holm L., (2022). Dali server: structural unification of protein families. Nucleic Acids Res. 50, W210–W215. 35610055 10.1093/nar/gkac387 PMC9252788

[42] Aderinwale T, Bharadwaj V, Christoffer C, Terashi G, Zhang Z, Jahandideh R, , (2022). Real-time structure search and structure classification for AlphaFold protein models. Commun. Biol 5, 1–12. 35383281 10.1038/s42003-022-03261-8 PMC8983703

[43] Bordin N, Sillitoe I, Nallapareddy MV, Rauer C, Lam SD, Waman VP, , (2022). AlphaFold2 reveals commonalities and novelties in protein structure space for 21 model organisms. bioRxiv. 10.1101/2022.06.02.494367. PMC9908985 36755055

[44] Dobson L, Szekeres LI, Gerdán C, Langó T, Zeke A, Tusnády GE, (2022). TmAlphaFold database: membrane localization and evaluation of AlphaFold2 predicted alpha-helical transmembrane protein structures. Nucleic Acids Res. 51, D517–D522. 10.1093/nar/gkac928 PMC9825488 36318239

